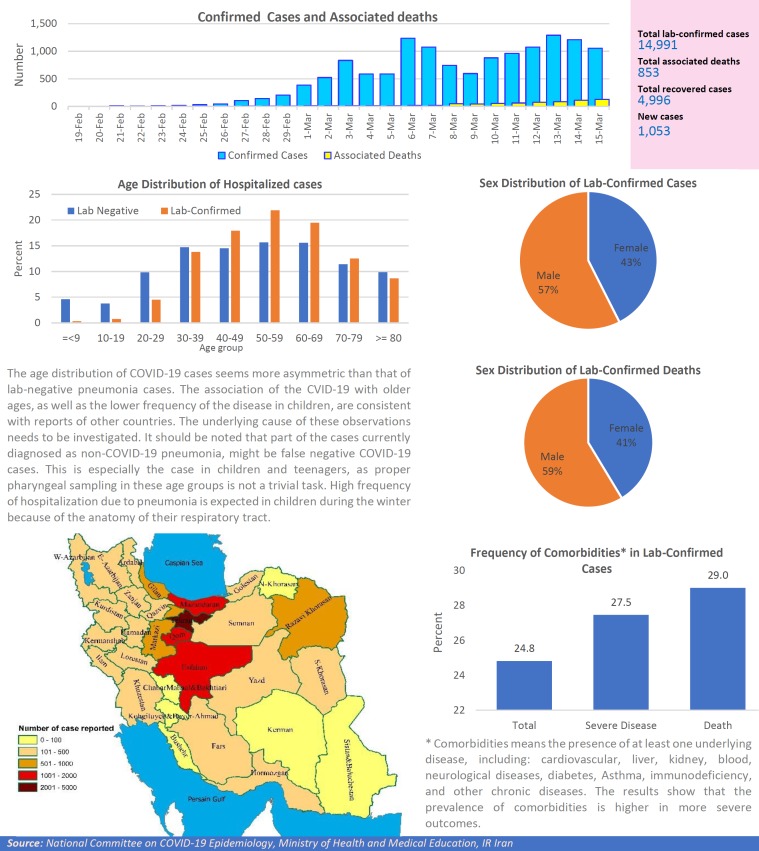# Daily Situation Report on Coronavirus disease (COVID-19) in Iran; March 16, 2020

**Published:** 2020-03-16

**Authors:** National Committee on COVID-19 Epidemiology, Ministry of Health and Medical Education, IR Iran

**Keywords:** COVID-19, severe acute respiratory syndrome coronavirus 2, Hospital Mortality, epidemiology, Pandemics, Health Information Exchange

## Abstract

After detection of the first confirmed cases of COVID-19 in Iran, the National Committee on COVID-19 Epidemiology in Ministry of Health and Medical Education was established. This Committee is official source of gathering, analyzing, and reporting the COVID-19 data in Iran. The data of all sources in the country including, medical care monitoring center (MCMC), Hospitals’ Information Systems (HIS), Laboratory portal, the data of the center for communicable disease control (MOH), as well as the data from community health centers are integrated and used in this regards. This factsheet contain daily situation report on coronavirus disease (covid-19) in Iran; March 16, 2020.

**Figure:**